# Demyelinating Disease following Anti-TNFa Treatment: A Causal or Coincidental Association? Report of Four Cases and Review of the Literature

**DOI:** 10.1155/2013/671935

**Published:** 2013-05-16

**Authors:** E. Andreadou, E. Kemanetzoglou, Ch. Brokalaki, M. E. Evangelopoulos, C. Kilidireas, A. Rombos, E. Stamboulis

**Affiliations:** 1st Department of Neurology, Athens National and Kapodistrian University, Aeginition Hospital, 11528 Athens, Greece

## Abstract

Tumor necrosis factor antagonists (anti-TNFa) are an established therapeutic option for several autoimmune and inflammatory bowel diseases. Despite their clinical effectiveness, neurological adverse events have been reported and literature data suggest a potential role of anti-TNFa in the induction of demyelination of the CNS. We present four patients treated with anti-TNFa who developed symptoms suggestive of CNS demyelination. The first patient, a 17-year-old male who received etanercept for psoriatic arthritis for eight months, presented with dysesthesias up to T4 level. The second patient, a 30-year-old male treated with adalimumab for three years due to ankylosing spondylitis, presented with right unilateral tinnitus. The third case, a 47-year-old female, received etanercept for four years because of psoriatic arthritis and developed persistent headache and left-sided face and head numbness. Finally, the fourth patient, a 57-years-old female treated with etanercept for six years due to ankylosing spondylitis, presented with difficulty in speech, swallowing, and ptosis of the right corner of the mouth. In all cases, brain MRI showed lesions suggestive of demyelination, while positive oligoclonal bands were detected in the CSF. Anti-TNFa treatments were discontinued and patients showed clinical improvement with pulsed intravenous corticosteroid therapy. CNS demyelination following anti-TNFa treatment represents a relatively rare but potential serious complication. Close follow-up and MRI monitoring of these patients is mandatory to elucidate whether the clinical manifestations represent adverse events occurring during anti-TNFa therapy or a first demyelinating episode.

## 1. Introduction

TNFa is a pleiotropic cytokine that plays a key role in host defense mechanisms and presents multifunctional proinflammatory properties [1–4]. Anti-TNFa agents have revolutionized therapy in rheumatoid arthritis, ankylosing spondylitis, psoriasis, psoriatic arthritis, juvenile polyarticular rheumatoid arthritis, and inflammatory bowel disease, with great success, regarding efficacy and safety [5, 6]. At present five anti-TNF agents have been authorized for clinical use [4]: the soluble TNFR2 (etanercept) and the 4 anti-TNF specific monoclonal antibodies (infliximab, adalimumab, golimumab, and certolizumab). They act by abrogating the soluble TNFa, thus preventing its binding on TNFR1/TNFR2 receptors [2]. 

Although anti-TNFa agents have been established as efficacious and relatively safe treatment with rare serious side effects [5], their increasing use during the last decade has revealed a variety of immune-mediated adverse events, the precise mechanism of which is not fully understood. Several potential risks such as predisposition to viral, fungal, and bacterial infections (especially tuberculosis), hemocytopenias, congestive heart failure, or development of T-cell lymphomas have been reported as well as injection/infusion site reactions [5, 7, 8]. Clinical signs of autoimmune disease, such as type 1 diabetes mellitus, lupus-like syndromes, psoriasis, and vasculitis may also arise in some patients [5, 9]. Moreover, numerous reports of emerging neurological complications have been described, the most frequent of which is either the development or the exacerbation of demyelinating conditions of the CNS [10–28]. However, there is a debate whether treatment with anti-TNFa unmasks preexisting multiple sclerosis (MS) or it induces de novo demyelination of the CNS. 

Herein we report four patients who developed neurological symptoms suggestive of CNS demyelination while undergoing anti-TNFa treatment and discuss the possible association of these clinical manifestations with the administration of anti-TNFa agents.

## 2. Report of Cases 

### 2.1. Case**  **1

A 17-year-old Caucasian male, with a history of juvenile onset psoriasis and a nine-month history of psoriatic arthritis, received etanercept for eight months. He presented with numbness of the left leg, which ascended to the left side of the trunk over the next three days. Several episodes of numbness of upper and lower extremities and body were reported over the past month of admission as well as an episode of retrobulbar pain of the right eye, lasting two days. According to his family history, his mother also received etanercept for psoriatic arthritis. An aunt from the maternal line and one from the paternal line also have psoriasis (Table 1).

The neurological examination revealed dysesthesias of the left leg and left abdominal region up to T4 level and brisk tendon reflexes of the lower extremities. Cranial nerves were intact. Routine blood tests were within the normal limits. Tests for rheumatoid factor (RA), antinuclear antibody titre (ANA), anti-double-stranded DNA (ds-DNA), anticardiolipin antibodies (ACA), and antiphospholipid antibodies (anti-*β*2 GPI) were unremarkable. 

**Figure 1:**
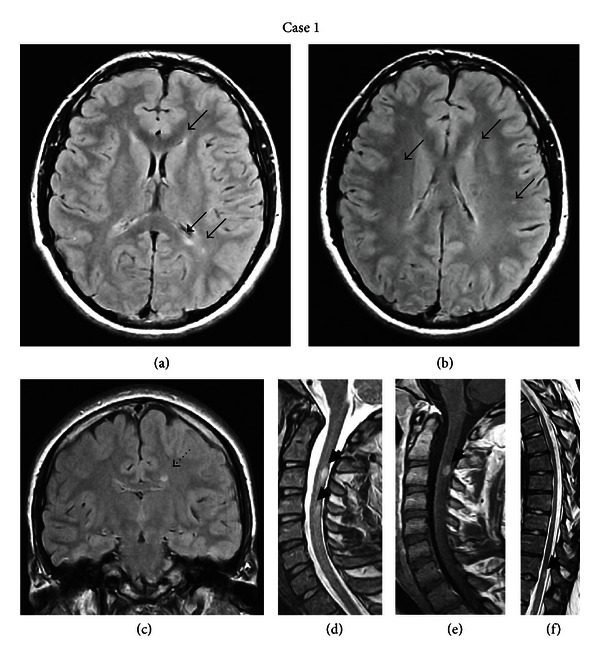
(a) and (b): Axial FLAIR brain MRI of the first patient demonstrating right subcortical and juxtacortical and left periventricular hyperintense lesions (black arrows). (c) Coronal FLAIR brain MRI showing a left periventricular hyperintense lesion (dotted black arrow). (d) Sagittal T2-weighted MRI of the cervical spine showing hyperintense lesions on C3 and C4-C5 level (black arrowheads). (e) The lesion on C3 level appears to be enhanced on T1-weighted image (black arrowhead). (f) Sagittal T2-weighted MRI of the thorasic spine demonstrating a hyperintense lesion on T11 level (black arrowhead).

Brain magnetic resonance imaging (MRI) showed periventricular and subcortical hyperintensities on T2-weighted images in both hemispheres without gadolinium enhancement on T1-weighted images (Figures 1(a), 1(b) and 1(c)). The cervical spinal MRI showed one non-enhancing lesion at C4-C5 level and one enhancing lesion at C3 level (Figures 1(d) and 1(e)). The thoracic spinal MRI revealed one non-enhancing lesion at T11 level (Figure 1(f)). Cerebrospinal fluid (CSF) examination revealed mild pleocytosis (15 lymphocytes/mm^3^), positive oligoclonal bands, and a raised IgG index. 

The treatment with etanercept was discontinued and the patient was treated with methylprednisolone pulse therapy (1 gr methylprednisolone/day iv for 5 days), followed by prednisone tapering. On discharge from the hospital, his neurological symptoms had almost completely resolved. 

At the follow-up visits at 3, 6, 9, 12, and 18 months later, the sensory symptoms had gradually subsided, and he was started on cyclosporine. Follow-up brain, cervical and thoracic spinal MRI at 6 months showed improvement: only two of the previous brain lesions were distinguished, none of the two cervical spinal lesions was enhancing, and the lesion on T11 was no longer detected. Repeated cerebral and spinal MRIs at 12 and 18 months were unchanged.

### 2.2. Case**  **2

**Figure 2:**
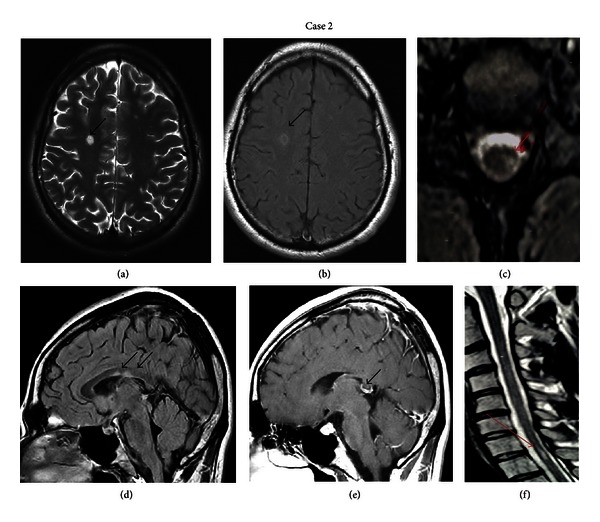
(a) Axial T2- and (b) T1-weighted brain MRI of the second patient showing subcortical hyperintense lesions, one with contrast enhancement (black arrows). (d) and (e) Sagittal T1 weighted with gadolinium brain MRI images showing enhanced corpus callosum lesions (black arrows). (c) Sagittal and (f) axial T2-weighted cervical spine MRI showing one hyperintense lesion (red arrows) on level C7-T1.

A 30-years-old Caucasian male was treated with adalimumab for three years due to HLAB27 negative ankylosing spondylitis. He presented with right unilateral tinnitus of two months duration. He also complained of fatigue and urinary urgency a few months after treatment onset. According to his family history, his father suffered from multiple sclerosis and his younger sister from psoriasis. The neurological examination at the time of admission showed extensor left plantar response, hyperactive deep tendon reflexes on upper and lower extremities, and absent abdominal reflexes. Routine blood analysis was unremarkable. Screening for autoantibodies (RA, ACA, ds-DNA, and *β*2GPI) was also unremarkable although ANA were mildly elevated (titre 1/160). The immunoglobulin IgA and IgG levels were also raised. MRI of the petrous bone was normal. Brain MRI showed subcortical and periventricular hyperintense lesions on T2-weighted and fluid attenuated inversion recovery (FLAIR) images, as well as lesions in the corpus callosum. Some of the lesions enhanced after gadolinium injection on T1-weighted images (Figures 2(a), 2(d), 2(e), and 2(f)). Cervical MRI showed one non-enhancing lesion at the C7-T1 level (Figures 2(b) and 2(c)). The visual and brainstem auditory evoked potentials were normal. The otolaryngologist assessment revealed reduced hearing acuity of the right ear. The CSF analysis yielded normal levels of glucose, raised protein (63 mg/dL), mild pleocytosis (10 lymphocytes/mm^3^), intrathecal synthesis of IgG and presence of oligoclonal bands.

The patient was treated with intravenous boluses of methylprednisolone (1 gr methylprednisolon/day iv for 5 days) followed by prednisone tapering and showed gradual clinical improvement. The treatment with adalimumab was discontinued, and at the clinical follow-up 3 months later, the patient was completely asymptomatic. The brain and cervical MRI were unchanged.

### 2.3. Case**  **3

A 46 year-old Caucasian female, with a history of psoriatic arthritis, for which she received etanercept for 4 years, presented with persistent left parietotemporal headache and numbness of the left side of the face and head for four months. She also mentioned urinary urgency and diminished libido the last few months. There was no family history of neurological disease. 

Neurological examination revealed left dissociated horizontal nystagmus and bilateral vertical nystagmus during the upward direction of gaze, right Hoffmann sign, and hyperactive deep tendon reflexes. Routine laboratory tests were within the normal limits, and the autoantibody tests were negative. A mild elevation of serum angiotensin converting enzyme (SACE) was noted (67.5 IU/L, normal rate: 23–57 IU/L); however, no pathological findings in the chest X-ray suggestive of sarcoidosis were found. 

**Figure 3:**
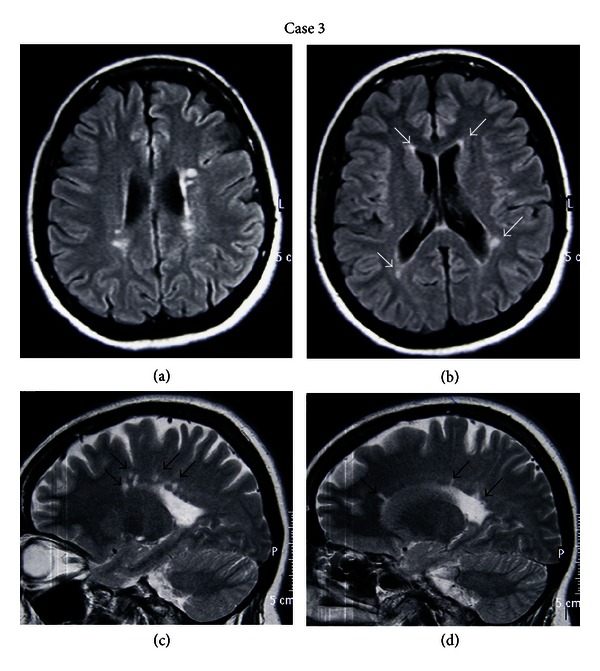
(a) and (b) Axial FLAIR brain MRI images of the third patient demonstrating multiple periventricular and subcortical lesions at the parietal and temporal lobes (white arrows). (c) and (d) Sagittal T2-weighted brain MRI images showing multiple lesions of the corpus callosum (black arrows).

Brain MRI revealed multiple periventricular white matter and temporal lobe lesions, without gadolinium enhancement (Figure 3). There were no lesions on the cervical spinal MRI. The CSF examination showed elevated IgG index and positive oligoclonal bands. 

She received methylprednisolone (1 gr methylprednisolone/day iv for 5 days) followed by prednisolone tapering and rapid clinical improvement was noted. The therapy with etanercept was stopped and at the follow-up after 3, 6, 12, and 18 months the patient was free of neurological symptoms. 

### 2.4. Case**  **4

A 57-years-old Caucasian female treated with etanercept for six years due to ankylosing spondylitis, presented with difficulty in speech, swallowing, and ptosis of the right corner of the mouth. Her past medical history was unremarkable except from hypothyroidism for which she was treated with thyroxine. No family history of neurological disease was mentioned.

**Figure 4:**
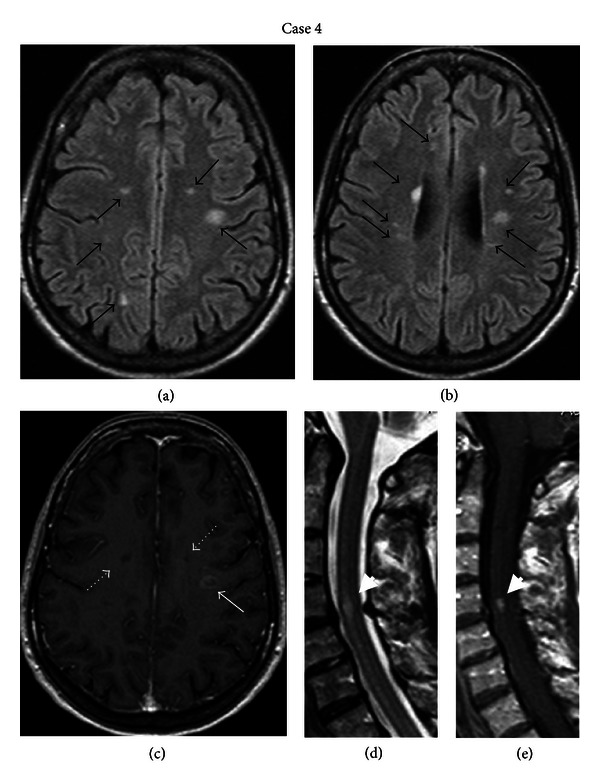
(a) and (b) Axial FLAIR brain MRI images of the fourth patient demonstrating multiple periventricular and subcortical lesions at the frontal, parietal, and temporal lobes (black arrows). (c) Axial T1-weighted brain MRI image showing gadolinium ring enhancement in one of the subcortical lesions (white arrow) and two subcortical black holes (white dotted arrows) (e) Sagittal T2- and (f) T1-weighted MRI of the cervical spine showing one hyperintense lesion at the C4-C5 level with gadolinium enhancement (white arrowheads).

The neurological examination at the time of admission revealed peripheral paresis of the right facial nerve, dysarthria, and mild surface dysgraphia. Routine blood analysis and screening for autoantibodies were unremarkable. Brain MRI revealed periventricular hyperintense lesions on T2-weighted and FLAIR images (Figures 4(a) and 4(c)), one of them in the subcortical area of the left parietal lobe with gadolinium enhancement (Figure 4(b)). Cervical MRI showed one hyperintense lesion at the C4-C5 level with gadolinium enhancement (Figures 4(d) and 4(e)). The CSF analysis yielded normal levels of glucose, raised protein (65 mg/dL), normal white blood count (4 lymphocytes/mm^3^), intrathecal synthesis of IgG, and presence of oligoclonal bands.

The treatment with etanercept was discontinued and the patient was treated with intravenous boluses of methylprednisolone (1 gr methylprednisolone/day iv for 5 days) followed by prednisone tapering and showed speech improvement. 

## 3. Discussion

In the presented cases, given the emergence of neurological symptoms and the detection of brain and spinal cord MRI lesions consistent with demyelination, a MS-like syndrome was suspected. According to the current diagnostic criteria for MS [29], the simultaneous presence of asymptomatic gadolinium enhancing and non-enhancing periventricular and juxtacortical lesions, as well as spinal cord lesions, and the presence of oligoclonal bands and raised IgG index in the CSF in the first, second, and fourth patient establish the diagnosis of MS. The third patient does not fulfill the criteria for MS thus far, since dissemination in time is yet to be proved. Nonetheless, the clinical manifestations, the morphology, and distribution of cerebral and spinal hyperintense lesions, the negative autoantibody tests, and the presence of oligoclonal bands in the CSF in all the presented cases argue for the diagnosis of demyelination of the CNS. As all four patients were receiving treatment with anti-TNFa compounds at presentation, a correlation was speculated. 

Several lines of evidence suggest that TNFa, which is secreted by microglia and macrophages in the CNS, has a direct role in the etiopathogenesis of demyelination and MS [30, 31]. There are two forms of TNFa: a transmembrane protein (tmTNF) and a soluble form (sTNF). Both interact with two distinct receptors, TNFR1 and TNFR2, but sTNF shows greater affinity with TNFR1. It seems that TNFR1, which contains a death domain, is responsible for the signaling of cell apoptosis. The TNFR2 function is more complicated. It does not have a death domain and seems to signal either proliferation of the cells or apoptosis, based on poorly understood pathways. Even less understood is the fact that both TNFa and TNFa antagonists can induce reverse signaling [4]. TNFa has been found to play a key role in myelin and oligodendrocyte damage in MS [30, 31]. While at the first stages of the disease, it is considered to be involved in demyelination, in later stages it is essential for remyelination [20–34]. TNFa was found elevated in serum and CSF of patients with progressive MS [5]. Moreover, TNFa levels were shown to correlate with disease severity [11, 15, 22]. Additionally, TNFa blockers showed beneficial effects in animal models of experimental autoimmune encephalomyelitis [3]. Based on these observations, anti-TNFa agents were subsequently suggested as potentially efficacious therapy for MS. Surprisingly, however, the clinical trials with infliximab and lenercept in MS patients revealed an increase in both relapse rate and MRI lesion load. Specifically, in an open-label phase I safety trial with infliximab, a humanized mouse anti-TNFa monoclonal antibody, in two patients with rapidly progressive MS, the treatment was associated with increased disease activity due to the appearance of new gadolinium enhancing lesions on MRI [10]. Furthermore, a randomized double-blind placebo-controlled multicenter trial in 168 remitting-relapsing MS patients with Lenercept, a recombinant TNFR I fusion protein, had to be halted prematurely as it revealed higher frequency and increased severity of exacerbations [11]. These studies suggested that anti-TNFa agents may potentially initiate or unmask an underlying demyelinating disease. Indeed, poor results of TNFa blockade in MS have been reported since then [12, 27]. 

Besides the effect of anti-TNFa therapy on definite MS, a relation between anti-TNFa agents and newly onset of MS or MS-like syndromes has been documented [13–26, 35–38]. It is unclear, however, whether these demyelinating events are coincidental or whether they are causally associated with the use of TNFa antagonists. Although to date, more than 500 cases of various neurological complications have been described in relation to anti-TNFa agents including CNS demyelination, Guillain-Barré syndrome, Miller Fisher syndrome, polyneuropathies, leukoencephalopathy [21, 35–44] and most of them indicate a causal association, the possible correlation between CNS demyelination and the TNFa blockade is still disputed. Moreover, the incidence of CNS demyelination following anti-TNFa therapy is presently unknown. A case control study in an administrative database cohort of more than 10.000 patients with rheumatoid arthritis suggested that anti-TNFa agents are associated with an approximately 30% increase in the risk of a demyelinating event [26]. On the other hand, other researchers have found that the incidence of MS during anti-TNFa therapy did not differ significantly compared with the general population [15, 39]. 

There are several matters that deserve special mention, such as the timing of the demyelinating event following exposure to anti-TNFa treatment and the resolution or recurrence of signs and symptoms after cessation of therapy. In the literature, the interval between the administration of anti-TNFa agents and the appearance of symptoms varies greatly. Most researchers have reported that the average time between treatment initiation and onset of neurological symptoms is about five months [17]. However, others have described advent of symptoms after long-term exposure (up to 4 years) to TNFa antagonists [35], as happened in three of our cases, and some researchers described an evolution of the newly appeared neurological syndrome despite cessation of the anti-TNFa agent [39]. Obviously, in these cases, the pathogenesis could be merely explained by the fluctuating nature of relapsing-remitting MS rather than the administration of anti-TNFa therapy [39]. In spite of this, the temporal relationship between the demyelinating symptoms and the anti-TNFa treatment [21], the complete remission of symptoms after the withdrawal of the compound [39], and a positive rechallenge phenomenon (improvement of symptoms on discontinuation of the anti-TNFa agent and reappearance or worsening of symptoms on reexposure to the agent) described in several cases [12, 40] argue for a causal association. A possible relation is reinforced by the fact that anti-TNFa drugs can induce other autoimmune diseases such as vasculitis, lupus and pulmonary fibrosis [34], as well as a rise of ANA, anti-dsDNA, and ACA titters [5, 43]. As regards our cases, the neurological complications developed 8 months, 3 years, 4 years, and 6 years, respectively, after the initiation of treatment. It is interesting to note, however, that symptoms had already appeared in three of our patients prior to their hospitalization (one month, 2.5 years, and 6 months, resp.), but they were not considered important by the patients and therefore they had not been previously reported. Obviously, an accurate estimation of the onset of symptoms in relation to the initiation of anti-TNFa compounds is not feasible when the symptoms are mild. Nonetheless, although a temporal relationship cannot be established in our cases, the clinical improvement after treatment cessation suggests a causal relation. 

Another issue to be addressed is the possibility of higher rate of demyelination among patients with autoimmune diseases due to their underlying genetic susceptibility for various immunological disorders including MS. It should be noted, however, that the incidence/prevalence of MS in individuals with autoimmune diseases [12, 22] as well as the frequency of other autoimmune diseases in patients with MS [45, 46] are still largely unknown. Nevertheless, it is possible that the anti-TNFa agents may be operating as a promoting factor in patients who are genetically predisposed to develop MS. Moreover, it must be taken into account that relatives of MS patients, especially first degree relatives, carry an increased risk of developing MS [45]. Given the positive family history of our second patient, he was more prone to develop MS. Moreover, the presence of symptoms prior to the initiation of treatment with the anti-TNFa agent supports the possibility of preexisting latent MS. However, this hypothesis cannot be substantiated, as neither neurological evaluation, nor imaging or CSF studies were performed prior to the administration of adalimumab. 

The mechanisms underlying the predisposition to demyelination or exacerbation of demyelination in patients treated with anti-TNFa agents are currently under investigation, and several theories have been proposed [17]. It has been postulated that anti-TNFa molecules do not penetrate the blood brain barrier (BBB), thus being unable to target the brain compared to other target organs [10]. The failure of TNFa antagonists to enter the CNS is a reasonable explanation for their inability to suppress demyelination induced by TNFa in MS. However, active demyelination renders the BBB permeable, which in turn facilitates the entry to CNS of autoreactive peripheral T cells that provoke demyelination. Therefore, it has been hypothesized that abrogation of the beneficial role of TNFa in remyelination might be associated with the reported exacerbation of MS [10, 15, 21, 33]. There is evidence suggesting that TNFR2 function is critical for the enhancement of remyelination with the use of anti-TNFa agents [30]. Additionally, it has been demonstrated that complete TNF inhibition in MS, including inhibition of the tmTNF-TNFR2 axis, causes serious adverse effects, whereas specific abrogation of TNFR1 is beneficial [29]. Under this light, TNFR2 agonists have been proposed as treatment of autoimmune diseases suggesting that TNFR2-mediated signaling can reduce inflammation and initiate repair [9, 30]. It should be mentioned that although these hypotheses seem likely to explain the failure of TNF inhibition in MS, they cannot adequately elucidate the onset of demyelination, as in our presented cases. Although the clinical manifestations in most reported cases of CNS impairment during treatment with anti-TNFa are consistent with demyelination, few patients meet the McDonald criteria for MS [23, 29]. As it has already been mentioned, it should be kept in mind that in these cases the appearance of demyelination following anti-TNFa therapy might be attributed to unmasking of clinically latent MS [12]. In spite of this, a male preponderance and an older age have been reported among patients receiving TNFa antagonists that develop demyelinating disease [22, 27, 39], an observation suggesting that this syndrome may differ from typical MS. These matters remain uncertain, requiring systematic and long-term monitoring of the affected patients. 

## 4. Conclusions

It is estimated that 2 million patients with various autoimmune disorders have been successfully treated with TNFa blockers so far [9, 34]. The reported cases of demyelination of the CNS during anti-TNFa therapy, however, raise questions about a possible causal association. 

Although several theoretical explanations of the possible relationship between TNFa blockade and demyelination have been proposed hitherto, none is considered adequate. The occurrence of demyelination in patients receiving anti-TNFa treatment, as happened in our patients, could be either attributed to the unmasking of a latent preexisting MS, to the emergence of new demyelinating episode (either MS or MS like), or finally to incidental coexistence of the two disorders [18]. Another question to be answered is whether demyelination during anti-TNFa agents actually meets the criteria for MS diagnosis or whether it constitutes a different MS-like syndrome. 

Although the number of individuals with emerging demyelinating events during anti-TNFa treatment appears to be small, the episodes may be clinically silent, posing a difficulty in assessing their true incidence rate. Additionally, inadequate follow-up of most of reported cases further reduces the likelihood of reporting new exacerbations and therefore establishing a definite diagnosis. Longer follow-up of the affected patients, including ours, is needed to address these issues thoroughly. 

Nevertheless, anti-TNFa treatment should be avoided in patients with MS or patients with a positive family history of MS [12, 19, 22, 40, 47], detailed neurological evaluation should be performed prior to TNFa blockers administration and close neurological monitoring during treatment. Moreover, clinicians should also be alert to distinguish symptoms suggestive of CNS demyelination, and in cases of high clinical suspicion, brain MRI scan should be performed even prior to treatment initiation [10, 18, 24]. Furthermore, in case of emerging demyelination during treatment, TNF*α* antagonists should be discontinued and close clinical and MRI monitoring should follow [12, 36].

## Figures and Tables

**Table 1:** Demographical, clinical and imaging data of the four cases.

Cases	1	2	3	4
Gender	Male	Male	Female	Female
Type of rheumatic disease	PA	AS	PA	AS
Duration of anti-TNFa treatment	8 months	3 years	4 years	6 years
Family history	PA (mother), Ps (aunts)	MS (father), Ps (sister)	—	—
Anti-TNFa	Etanercept	Adalimumab	Etanercept	Etanercept
Age at onset of symptoms	17 years	30 years	46 years	57 years
Symptoms	Dysesthesias of left lower limb, T4 level	Right unilateral tinnitus, urinary urgency	Left parietotemporal headache, paresthesias of the left side of the face and head, urinary urgency, diminished libido	Peripheral paresis of right facial nerve, dysarthria, mild surface dysgraphia.
CNS MRI	Hyperintensities (1 Gd+ lesion at C3 level)	Hyperintensities (>5 lesions showed Gd+)	Hyperintensities (no Gd+ lesions)	Hyperintensities (1 Gd+ lesion at left parietal lobe and 1 at C4-C5 level)
OB	Yes	Yes	Yes	Yes
Evolution	MS	MS	Monophasic demyelinating event	MS

PA: psoriatic arthritis, AS: ankylosing spondylitis, Ps: psoriasis, anti-TNFa: tumor necrosis factor alpha antagonist, MRI: magnetic resonance imaging; OB: oligoclonal bands, MS: multiple sclerosis, and Gd+: gadolinium enhancement.
